# Novel nano composites from *Citrus limon* and *Citrullus colocynthis* agricultural wastes for biomedical applications

**DOI:** 10.1038/s41598-024-67423-w

**Published:** 2024-07-28

**Authors:** Nagwa A. Kamel, D. A. Wissa, Salwa L. Abd-El-Messieh

**Affiliations:** 1 Microwave Physics and Dielectrics Department, Physics Research Institute, National Research Centre, Giza, Egypt; 2 Solid State Physics Department, Physics Research Institute, National Research Centre, Giza, Egypt

**Keywords:** *Citrus limon*, *Citrullus colocynthis*, Polymer nanocomposite, Biofillers, Dielectric spectroscopy, Biocompatibility, Biophysics, Materials science

## Abstract

In recent years, academic and industrial research has focused on using agro-waste for energy and new material production to promote sustainable development and lessen environmental issues. In this study, new nanocomposites based on polyvinyl alcohol (PVA)-Starch using two affordable agricultural wastes, *Citrus limon* peels (LP) and *Citrullus colocynthis* (Cc) shells and seeds powders with different concentrations (2, 5, 10, and 15 wt%) as bio-fillers were prepared. The nanocomposites were characterized by Dielectric Spectroscopy, Fourier-Transform Infrared (FTIR), Scanning Electron Microscopy (SEM), Transmission Electron Microscopy (TEM), and water swelling ratio. The antimicrobial properties of the nanocomposites against *Escherichia coli*, *Staphylococcus aureus*, and *Candida albicans* were examined to investigate the possibility of using such composites in biomedical applications. Additionally, the biocompatibility of the composites on human normal fibroblast cell lines (HSF) was tested using MTT (3-[4,5-dimethylthiazol-2-yl]-2,5 diphenyl tetrazolium bromide) assay. The results demonstrate that the filler type and concentration strongly affect the film's properties. The permittivity ε′, dielectric loss ε″ and conductivity σ_dc_ increased by increasing filler content but still in the insulators range that recommend such composites to be used in the insulation purposes. Both bio fillers control the water uptake, and the samples filled with LP were more water resistant. The polyvinyl alcohol/starch incorporated with 5 wt% LP and Cc have antimicrobial effects against all the tested microorganisms. Increasing the filler content has a negative impact on cell viability.

## Introduction

For over 50 years, biodegradable polymers have been utilized in a wide range of applications, including medical implants, wound dressings, tissue regeneration, enzyme immobilization, and controlled drug delivery^1–3^. From these polymers, blends and composites from polyvinyl alcohol and starch exhibit a wide range of adaptable physical properties as well as other beneficial qualities at a reasonable cost and rate of biodegradation and excellent compatibility between the two polymers^4,5^. To further improve their properties, some techniques, such as cross-linking and incorporation of fillers or fibers, can be applied^6^.

Fruit skins naturally contain affordable, non-toxic, and ecologically friendly fibers, giving them distinct advantages over other materials^7^. Recent research has concentrated on finding ways to utilize these agricultural wastes because they have a high concentration of lignocellulosic matter, which often contains natural flavonoids with high health-promoting outcomes^8,9^.

Pomegranate peel (PGP) used as a reinforcing and antibacterial ingredient in starch-based films. The films have good antibacterial properties, and PGP enhances the mechanical properties of the films. The prepared composites can be utilized as edible films and food packaging material^10^. Kamel et al. used banana peel powder as reinforcement for chitosan. The antimicrobial properties of these composites were promising and recommended for use as a wound covering. Many other fruit peels were studied, such as oranges, tomatoes, pineapple, or watermelon^11–14^.

Lemon peels are considered byproducts in the manufacturing process of lemon juice; they contain two types of tissues: flavedo (outer layer) and albedo (inner layer). The most significant elements of lemon peels include protein, pectin, cellulose, hemicellulose, lignin, pigments, and bioactive compounds such as essential oils, polyphenols, vitamin C, and flavonoids^15^. The thermal stability and antioxidant activity of PVA/Starch films incorporated with lemon peel powder with concentrations 1, 2, 4, and 8 by wt were investigated by Pınar terzİoğlu and Yusuf Sicak. The study's results pointed to the enhancement of the properties of the films. They suggested the usage of such composites in food packaging to preserve foods susceptible to oxidation^16^.

Pınar terzİoğlu Fatma Nur Parin reported enhanced UV-light barrier properties and mechanical properties of PVA-starch composites by adding lemon peel powder. These composites were valuable for packaging applications^17^.

*C. colocynthis* L. is a medicinal plant from the family of Cucurbitaceae ^18^. It is a melon species grown in West Africa for its edible oil, protein, carbohydrates, and fiber-rich seed^19^. For ages, traditional medicine has employed the fruits of *C. colocynthis* as a treatment for hemorrhoids and diabetes. Additionally, treatments for bronchitis, ulcers, fever, anemia, and breast irritation^20^. Cucurbitacin, flavonoids, alkaloids, and phenolic acids are the primary active components of *C. colocynthis*, and recent pharmacological research has demonstrated that these compounds have antioxidant, anti-pathogenic microorganism, and anti-cancer effects^21,22^. The chemical characterization, antioxidant activity, and cytotoxic effect against colon cancer and breast cancer cell lines of ethanolic extract of *C. colocynthis* were performed by Bourhia et al. the study indicated the positive effect of the seed extract and suggests its using to fight against cancer and free radicals^23^.

This study compares the effect of using two affordable agro-wastes, lemon peel powder, and dry Colocynthis powder, in different concentrations as reinforcement fillers for PVA/Starch blends. The study involved structural, dielectric, antimicrobial properties, cytotoxic effects on skin fibroblasts, and swelling properties. The study aimed to evaluate the nanocomposites used in different biomedical applications such as anti-aging, wound healing and or skin patches.

## Materials and Methods

### Materials

PVA is supplied by Quali-kems from India. Wheat starch (St) provided by Fluka Company from USA. Sigma-Aldrich provides Glutaraldehyde (50%) from Germany, and Glycerol is provided by Fisher from Germany.

Test organisms: The strains used in microbiological analysis are gram-negative bacteria: Escherichia coli (ATCC 25922). Gram-Positive bacteria: Staphylococcus aureus (ATCC 6538). Pathogenic yeast: Candida albicans (ATCC 10231).

### Methods

#### Collection and preparation of the fillers

Lemon (*Citrus limon*) was collected from the Egyptian markets. Then the lemon was washed thoroughly with water, peeled off, the peels dried in a hot air oven (70 °C) overnight, and finally grinded into fine powder.

*Citrullus colocynthis* was purchased from Egyptian markets. The plant samples were cleaned with water and allowed to dry in the air for two weeks. The dried fruit (the shell and seeds) homogenized with an electric grinder to fine powder, and stored in sealed flasks for further use^24^.

Figure S1 is a representation of the whole fruit and the used parts from it.

#### Preparation of the PVA/starch blend

Typically, the casting procedure is used to create particular PVA/starch samples at a ratio of (50:50) wt%. Firstly, 3.5 g of PVA was dissolved in 75 ml of distilled water at 95 °C for 30 min to create a PVA solution. Next, for the PVA/Starch blend, mix starch, and glycerol at a ratio (70/30) wt% with water by using a magnetic stirrer for 10 min; following this, as a starch crosslinking agent, glutaraldehyde was applied at a concentration of 10% (based on starch weight). After that, starch was added to the PVA solution while stirring continuously to create a homogenous mixture. The produced mixture solution was put to clean Petri dishes and air-dry overnight. After the films were taken out of the Petri dishes and placed in vacuum desiccators to ensure that all of the water had been completely removed, they were used for further investigation^25,26^.

#### Preparation of LP or Cc/PVA/starch reinforced nanocomposites

0.07gm (2%) of LP or Cc was added to the PVA/starch solution and stirred with a magnetic stirrer for two hours To achieve a homogeneous distribution. Ultimately, the mixture was poured onto clean, dry Petri dishes and heated in an oven for 24 h at 60 °C to create the film. The previously described process was repeated for 5, 10, and 15 wt%, and it is the same method employed for creating blend film from LP or Cc with PVA/starch^27^.

### Techniques

#### Transmission electron microscope (TEM)

The particle size of the utilized bio-fillers was measured using TEM model: JEM-HR 2100 and accelerating voltage 200 kV, Japan.

#### Fourier-transform infrared (FTIR)

The spectrum was recorded with a JASCO FT/IR 300 E FTIR Spectrometer (Tokyo, Japan).

#### Scanning electron microscope (SEM)

Polymer nanocomposites were studied by SEM/EDX, Philips XL30, from Japan.

#### Dielectric measurements

The capacitance C and loss tangent tan δ were measured in the frequency range of 100 kHz to 120 MHz with the LCR HiTESTER 3535 Model. The MC-100 Dielectric Cell was used for such measurements. The permittivity ε′ was calculated from the well-known relation ε′ = C/Co where C is the capacity in the presence of the sample, and Co is that in the presence of air. The dielectric loss ε″ was obtained from the multiplication of the ε′ values by the loss tangent tan δ.

#### Electrical conductivity measurements

The volume resistivity ρ was measured by Super Meg Ohm Meter Hioki SM7110 (Hioki, Japan). The samples were placed in shielding box type Hioki SME3811 with outer and inner electrode diameters of 24 mm and 19 mm, respectively. This shield box is guarded and tuned for applying DC potential up to 1000 V. The Super MegOhm Meter Hioki SM7110 can measure small currents up to 1pA peco Amperes in resolution of 1fA femto Amperes. From which the electrical conductivity σ was calculated. The electrical conductivity σ was calculated from the measured resistivity ρ according to the well-known relation σ = 1/ρ.

#### Swelling ratio

Film squares of size 1 cm × 1 cm were used for the water swelling test. The film squares were dried in an oven until constant weight was reached (W_i_) and placed in petri dishes. 50 ml distilled water was then poured into each dish. After 60 min and 120 min of immersion at 37 °C, the excess water was removed by gently wiping film squares with filter paper, and the weights were recorded (W_f_). The water swelling percent is calculated as follows;

1 $${\text{Swelling }}\left( \% \right) \, = ({\text{ W}}_{{\text{f}}} - {\text{ W}}_{{\text{i}}}) /{\text{Wi }} \times 100$$

where: Wi and W_f_ are weights of films, initially and after time t, respectively.

#### Microbiological analysis

Using the shake flask method, the antimicrobial activity of specific samples was tested against two types of pathogenic bacteria: Gram-positive (*Staphylococcus aureus*), Gram-negative (*Escherichia coli*), and pathogenic yeast (*Candida albicans*). The reduction of colony forming units (CFU) was determined by measuring the optical density (OD) at 600 nm.

Using this method, the pathogenic strains' inoculum size was prepared from fresh working stoke cultures and adjusted to approximately 0.5 McFarland standard (1.5 × 108 cfu/ml). Additionally, 25.0 μl of both bacterial and fungal suspensions were poured under sterile conditions into 100.0 conical flasks containing 20.0 mL of a nutrient broth medium (NB). Each sample was applied separately on each of these inoculated flasks, and the suspensions were incubated for 24 h at 37 °C with 150.0 rpm rotating shaking conditions^28–30^.

The antimicrobial activity was measured throughout the relative [OD (%)] reduction of these pathogenic strains present in flasks containing the treated samples compared to the control flasks that contained pathogenic strains only without any treatments. All results were expressed according to the following equation:

2 $${\text{Relative }}\left[ {{\text{CFU}}\;{\text{ Reduction }}\left( \% \right)} \right] \, = \, \left( {{\text{A}} - {\text{ B }}/{\text{A}}} \right) \, \times { 1}00$$

where: A: The CFU of pathogenic strains only without any treatments present on the control flask. B: The CFU of pathogenic strains on the flask contains the treated sample.

#### Cytotoxic effect on fibroblast cell lines (HSF)

The mitochondrial-dependent reduction of yellow MTT (3-(4,5-dimethylthiazol-2-yl)-2,5-diphenyl tetrazolium bromide) to purple formazan was used to measure the viability of the cells. The following procedures were performed in a sterile environment with a Class II A2 Laminar Flow Biosafety Cabinet (Manufactured by Labconco).

The cells were suspended in DMEM medium, 1% antibiotic–antimycotic mixture (10,000 U/ml Potassium Penicillin, 10,000 µg/ml Streptomycin Sulfate, and 25 µg/ml Amphotericin B), 1% l-glutamine, and 5% fetal bovine serum at 37 °C by using the CO_2_ incubator (Sartorius stedium, biotech) under 5% CO_2_.

After batch culture for ten days, cells were seeded at a density of 10 × 10^3^ cells /well in freshly prepared growth medium in 96-well plastic plates. The plates were then kept at 37 °C for 24 h under 5% CO_2_, either without any drugs (negative control) or with various drug concentrations to obtain the final concentration of (1000, 500, 250, 125, 62.5, 31.25, 15.625, 7.812 µg/ml^31,32^.

Next, the absorbance was determined at 595 nm using a reference wavelength of 620 nm and a microplate multi-well reader (Bio-Rad Laboratories Inc., model 3350, Hercules, California, USA.

3 $${\text{Viability }} = {\text{ absorbance}}\;{\text{ of}}\;{\text{ drug }}/{\text{ absorbance}}\;{\text{ of}}\;{\text{ control }} \times { 1}00$$

4 $${\text{Cytotoxicity }} = { 1}00 - {\text{ viability}}$$

## Results and Discussion

### Transmission electron microscope

**Figure 1:**
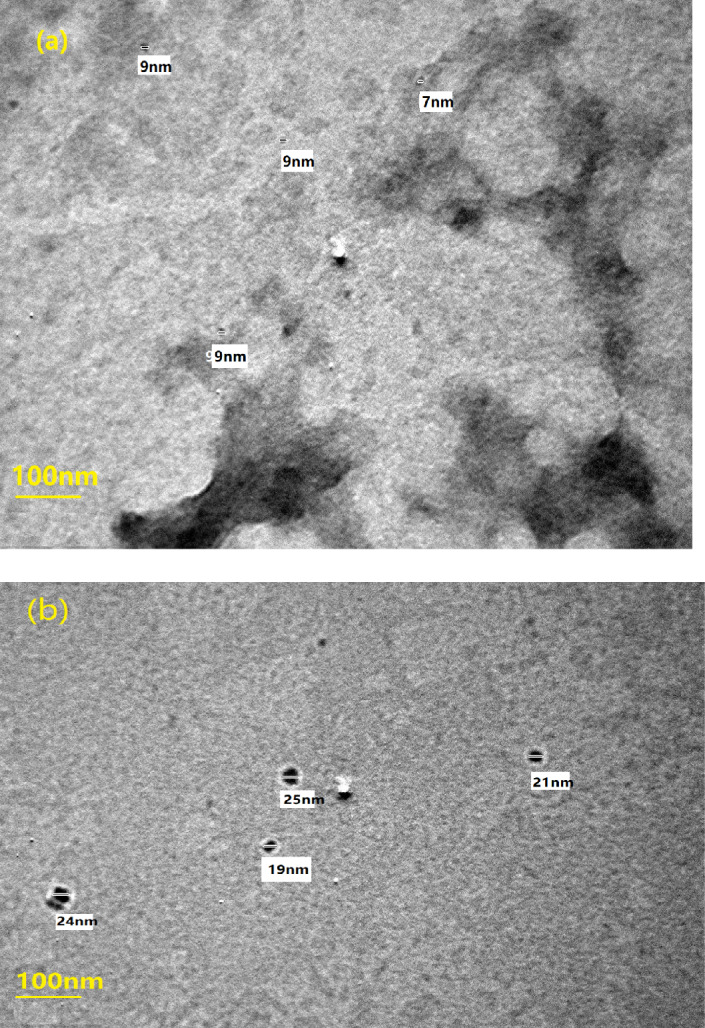
TEM images of (**a**) *Citrus limon* peels, (**b**) *Citrullus colocynthis* particles.

Figure 1 shows the TEM images of lemon peel powder (LP) and crushed *Citrullus colocynthis* (Cc) particles. The micrograph shows ultrafine particles in the nano range with diameter ranges 7–9 nm for lemon peel particles and from 19 to 25 nm for Cc.

### Fourier-transform infrared

FTIR analysis is an essential tool for understanding the interactions at the interfaces of the matrix and the incorporated fillers. The FTIR spectrum of the PVA/starch (Blank) polymer blend is displayed in Fig. S2. The assignment of the spectrum is listed in Table 1.

**Table 1:** Blank PVA/starch main bands^33–35^.

3281	Stretching vibration of the OH groups of both starch and PVA
2919	C–H group present in the starch and PVA
1715, 1730	C=O bond of carboxylic acid group
1425	CH_2_ bending
1373	Deformation vibration of the CO groups stretching
1246	CH vibration
1078	Vibration of CO bond in C–O–H
926	C–H rocking of PVA
848	Stretching vibration of C–C groups

**Figure 2:**
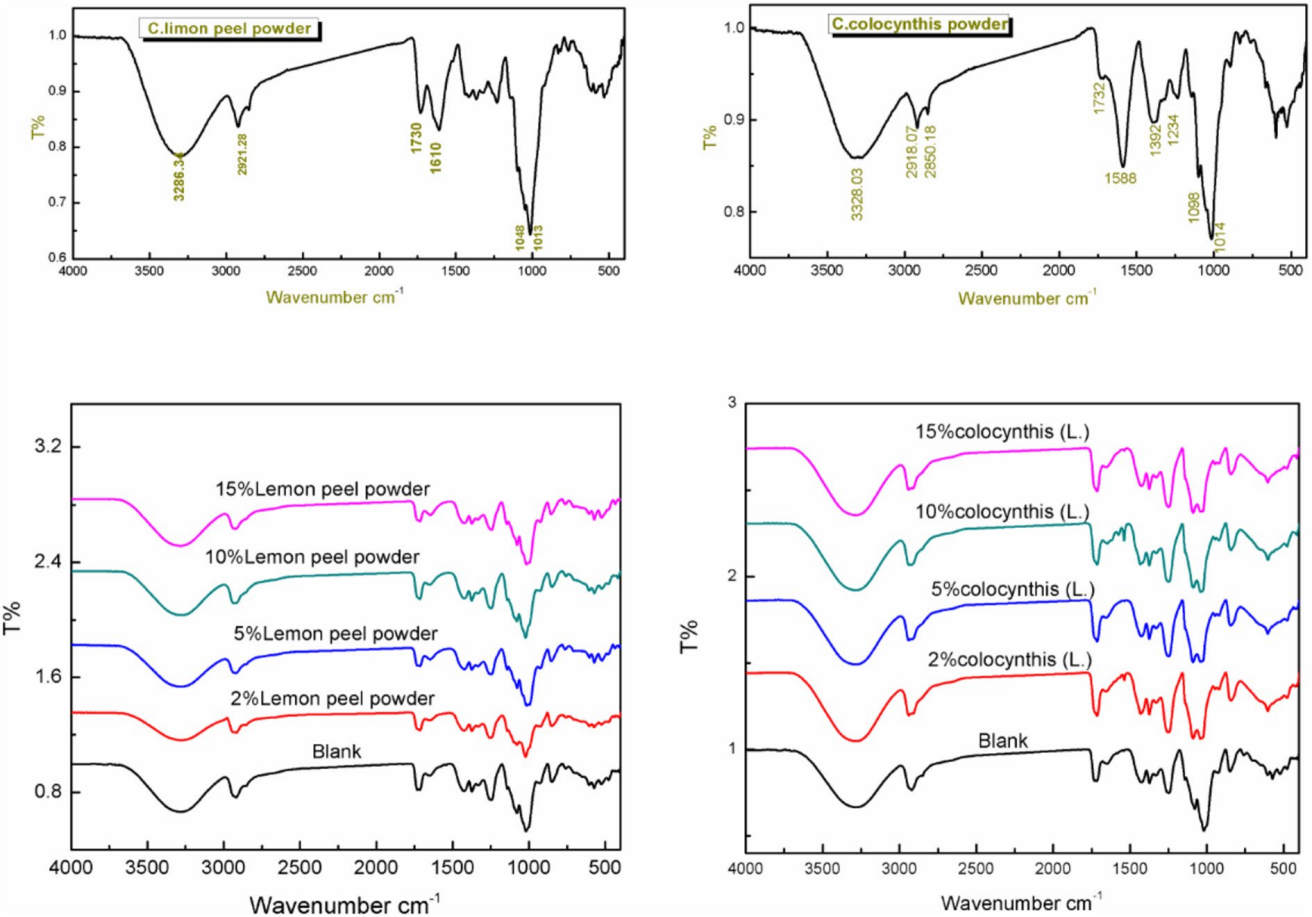
FTIR spectra of lemon peel powder (LP) and PVA/starch/LP nanocomposites (at the left), *Citrullus colocynthis* powder (Cc) and PVA/starch/Cc nanocomposites (at the right).

From Fig. S2, the presence of both polymers was evident in the blank polymer blend's spectrum. Figure 2 represents the FTIR spectra of the nanocomposites along with individual LP and Cc nanofillers.

### FTIR analysis for LP and PVA/starch/LP nanocomposites

The spectrum of the LP, and the main bands identification are 3286 cm^−1^refered to hydroxyl groups OH of macro molecular association (pectin and cellulose). 2921 cm^−1^: asymmetric stretching of –CH group. 2850 cm^−1^ symmetric stretching vibration of –CH group. 1608 cm^−1^; stretching vibration of carboxylate ions (COO–) of pectin. 1730 cm^−1^: stretching vibration of ester carbonyl group(C=O). 1020–1300 C–O group. The spectrum is in accordance with others reported for lemon peel^36,37^.

Some changes were observed in the spectra after incorporating LP into the PVA/starch matrix. It could be observed that the peak of hydroxyl groups at 3281 cm^−1^ has shifted to higher wave numbers, and their intensities were lowered compared to the spectrum of the blank. The 759 cm^−1^ peak shifted to a higher wave number. There were also changes in the intensity of nearly all other peaks. These changes may be attributed to modified PVA/starch association or the formation of new hydrogen bonds between the PVA, starch, and lemon peels^17,38^.

### FTIR analysis for Cc and PVA/starch/Cc nanocomposites

The spectrum of Cc revealed the presence of 3328 cm^−1^ peak referred to –OH groups of alcohol and phenol. 2918 cm^−1^ asymmetric stretching of –CH group. 2850 cm^−1^ symmetric stretching vibration of –CH group. 1588 cm^–1^: Phenol ring.1393 cm^−1^: stretching vibration of –COO. 1234 cm^−1^ C=O stretching vibration. 1014 cm^−1^ C–O stretching of carboxylate groups. 894.48, 832.73 cm^−1^ C–H.

After incorporation of Cc in the PVA/starch composites, the spectra revealed the presence of numerous changes; there was a decrease in intensities of some bands such as 3281 cm^−1^, 1715, 1647, 1377and 1246 cm^−1^ and the bands at 1078, 1019 and 924 cm^−1^ were shifted to higher wave number. The appearance of a new band at 1538 cm^−1^ which present in the spectrum of the Cc due to the phenol ring. All these changes predict the presence of new bonds that takes place between Cc and the PVA/starch blend. These bonds are mainly due to the presence of many functional groups in the Cc structure, as seen in the spectrum in Fig. 2.

### Swelling ratio

In the swelling test, after immersion of the samples in distilled water at 37 °C, unfilled PVA/starch film dissolved in water within the first hour, which indicates poor resistivity to water. This was expected and consistent with previous studies since PVA and starch are hydrophilic polymers^25^.

**Figure 3:**
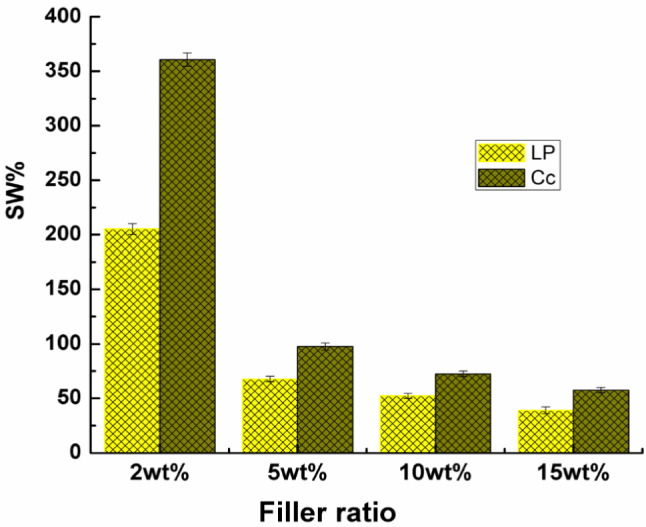
Comparison between swelling ratios of the nanocomposites containing different concentrations of the nano-fillers.

The effect of the type and concentration of the nano-fillers on the water swelling behavior of PVA/starch nanocomposites loaded with LP and Cc is represented in Fig. 3. From the figure, the swelling percentage decreases by increasing the LP and Cc filler concentration, improving the filled nanocomposites' water resistance. Moreover, it is also observed that, the nanocomposites containing LP were more resistant to water absorption than that containing Cc.

The controlled swelling behavior of the filled films may be linked to the crosslinking effect of the fillers and PVA/starch matrix, which is confirmed by FTIR results, thus depreciating the possibility of water uptake.

### Scanning electron microscope

**Figure 4:**
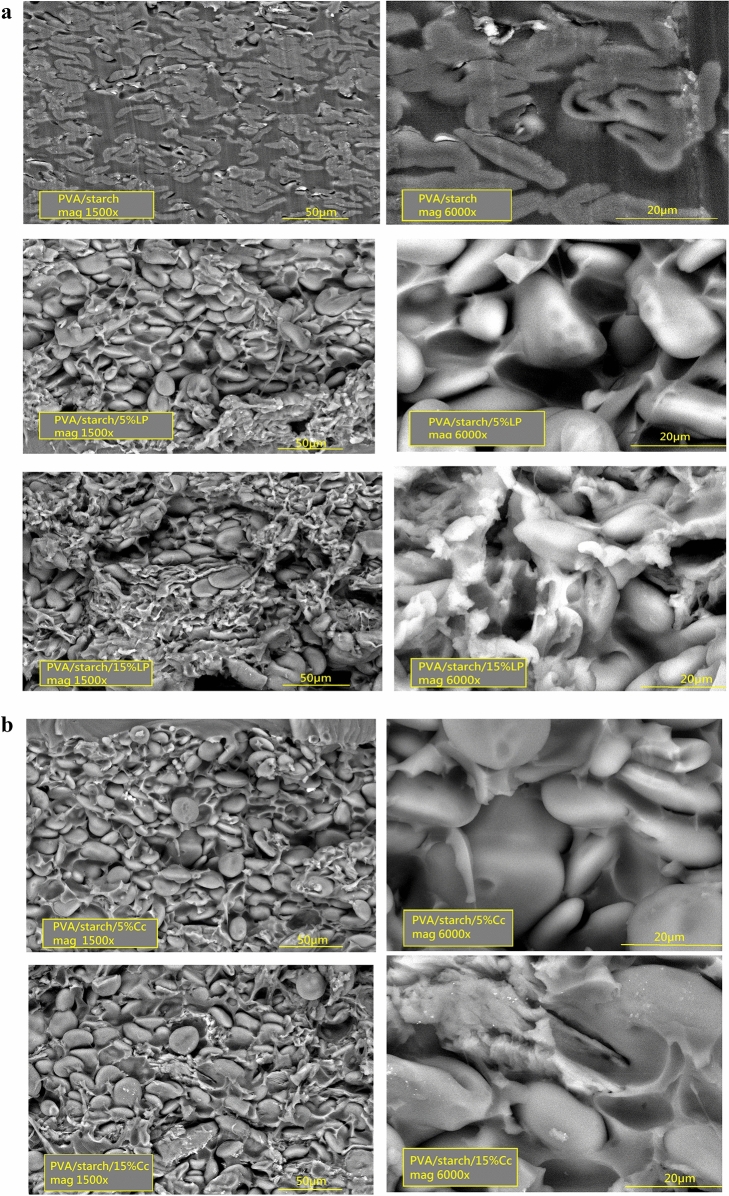
(**a**) SEM micrographs of the cross-sections of PVA/starch containing 5 & 15 wt% LP mag 1500 and 6000 ×. (**b**) SEM micrographs of the cross-sections of PVA/starch containing 5 & 15 wt% Cc mag 1500 and 6000 ×.

SEM micrographs of PVA/starch, PVA/starch/LP, and PVA/starch/Cc nanocomposites with filler concentrations 5 and 15 wt% for the sake of brevity were presented in Fig. 4a and b with two magnifications. The pristine PVA/starch blend exhibited a relatively heterogeneous structure, which suggested intermediate miscibility of PVA with starch in the blend.

After incorporation of the nano-fillers, LP and Cc dramatic changes in the blend films were observed. Increasing the concentration of the filler makes the matrix denser and more compact, which appears clearly in the nanocomposites containing 15 wt% for both fillers as an interlinked structure. This is considered a reasonable interpretation for the decrease in the water uptake by increasing the filler content, and the high swelling of the unfilled polymer blend is a result of its loose network compared to the reinforced ones.

### Dielectric measurements

**Figure 5:**
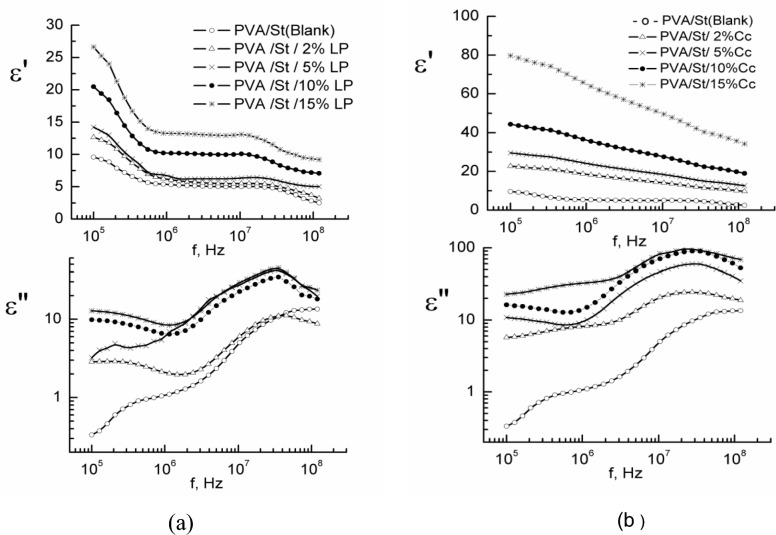
ε′ and ε″ V.s applied f, for (**a**) PVA/starch/LP nanocomposites and (**b**) PVA/starch/Cc nanocomposites.

The permittivity ε′ and dielectric loss ε″ were measured over a frequency range 10^5^ to 10^8^ Hz at a room temperature of about 25 °C. The obtained data are given in Fig. 5a and b.

The ε′ values of a matter can be a good indicator of the possibility of its capacity to store up energy in the incidence of the electric field. Figure 5a and b show that ε′ are high at low frequencies for nanocomposites containing both fillers. The increase in ε′ values at lower frequencies could be explained according to the space charge effects^39,40^ and the interfacial polarization^41^. This rise in governed space charge polarization, due to the formation of the confidential mobility of the bound dipole carriers, is reliable for the formation of orientation polarization^42^. This increase was quiet down and became linear, showing no further logical change at high-frequency ranges.

The ε″ explains the attendant loss or the dissipation of power^43,44^. With the same condition as ε′, the dielectric loss ε″ for LP and Cc nanocomposite systems was measured as a function of the applied f, and the data are pointed up graphically in Fig. 5a and b. The ε″ is taken as an indicator of the losses in energy due to the survival of the movement of dipoles between the PVA/starch matrix and the used filler. At that frequency range, the losses may be due to the moving dipoles due to the presence of filler^45,46^. To recognize the effect of these fillers on both ε' and ε″ and how they are exaggerated either by the type or content of the used fillers, both values are demonstrated in Fig. S3 at fixed frequency f = 100 kHz. That figure reflects that both ε′ and ε″ increased by increasing filler content; in addition, nanocomposite containing Cc as a filler possess higher values of ε′ and ε″ than those of LP-filled ones.

Figure 5a and b show that the curves concerning εʺ and the applied f are so convoluted and are a sign of the presence of extra than one relaxation procedure. So, it was worthwhile to analyze such curves to obtain the various relaxation processes. The analyses of such curves after subtraction of the losses due to the electrical conductivity were done using two Frohlich terms with distribution parameter P = 4 and Havriliak Negami function according to the equations given elsewhere^47^. An example of the analyses for PVA/starch/ 10% LP was given in Fig. S4a. The obtained data of both τ_1_ and τ_2_ are given in Fig. S4b. The obtained relaxation times τ_1_ and τ_2_ could be ascribed to some local molecular motions of the side chain and attached groups rather than the main chain motion as it is expected to be frozen since the measurements were carried out at 25 °C, i.e., lower than the glass transition T_g_ of the polymers under investigations^48,49^.

### Electrical conductivity

**Figure 6:**
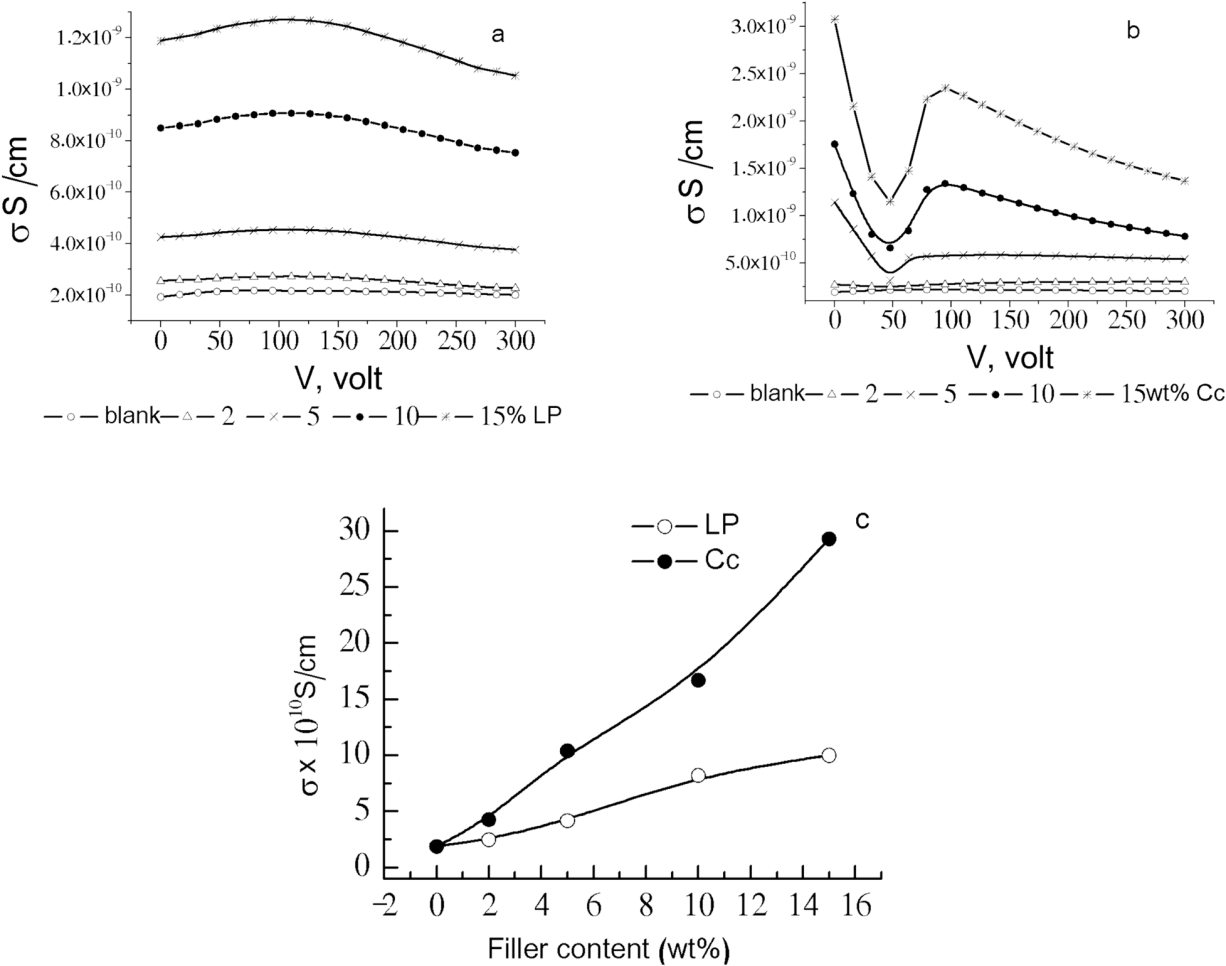
σ versus the applied volt for (**a**) PVA/starch/LP, (**b**) PVA/starch/Cc nanocomposites, (**c**) σ_dc_ versus filler content.

The calculated electrical conductivity σ was illustrated graphically versus the applied voltage V for both PVA/starch/LP and PVA/starch/Cc nanocomposites, Fig. 6a and b.

The Ohmic dc conductivity σ_dc_ was calculated from the linear relationship between σ_dc_ and the applied volt at low voltage values. The obtained data are illustrated graphically versus filler content in Fig. 6c. σ_dc_ values increased by increasing filler concentration for both fillers and Cc values were higher than composites containing LP.

Figure shows that σ_dc_ values are in the order of 10^–10^ S/cm, which highly recommends such composites to be used for insulating purposes.

### Cytotoxicity effect on fibroblast cell lines (HSF)

Cytotoxicity testing is a favorable first step to assure the biocompatibility of a biomaterial. In this study, despite *Citrullus colocynthis* being a herbal medicine, it has some toxic effects. Shafaei et al. investigated the toxic effects of *C. colocynthis* on albino rats. The rats given seed extract at doses of 100 or 200 mg/kg/day showed relatively mild intestinal damage. Unlike seed extract, *C. colocynthis* pulp extract has the potential to be lethal to rabbits. Thus, for medicinal applications, seed extract might be the best option^50^.

This study performed and suggested these new composites for topical applications so the toxicity study was of great concern.

The samples were tested against the Normal Human Skin Fibroblast. Samples concentration range between (1000 to 31.25 µg/ml) using MTT assay.

Figure S5 represents the viability versus different concentrations for 2 and 5 wt% LP-loaded nanocomposites for the sake of brevity, and Table 2 displays the viability, cytotoxicity, and IC_50_ of the nanocomposites containing LP.

**Table 2:** Cell viability, cytotoxicity, and IC50 for PVA/starch/LP nanocomposites at 125 µg/ml sample concentration.

	PVA/starch	PVA/starch/2%LP	PVA/starch/5%LP	PVA/starch/10%LP	PVA/starch/15%LP
Viability%	57.1	77.5	78.28	65.8	59.8
Cytotoxicity%	42.9	22.5	21.72	34.2	40.2
IC50	505.5784	892.2451	727.854	546.800	453.2984

IC50 (concentration of the sample which causes the death of 50% of cells in 48 h).

Figure S6 represents the viability versus different concentrations for 2 and 5 wt% Cc-loaded nanocomposites for the sake of brevity, and Table 3 displays the viability, cytotoxicity, and IC_50_ of the nanocomposites containing Cc.

**Table 3:** Cell viability, cytotoxicity and IC_50_ for PVA/starch/Cc nanocomposites at 125 µg/ml sample concentrations.

	PVA/starch	PVA/starch/2%Cc	PVA/starch/5%Cc	PVA/starch/10%Cc	PVA/starch/15%Cc
Viability%	57.1	70.5	69.07	44.2	40.1
Cytotoxicity%	42.9	29.5	30.93	55.8	59.9
IC_50_	505.5784	714.9374	712.5519	465.6562	318.1188

IC_50_ (concentration of the sample which causes the death of 50% of cells in 48 h).

The results indicated that the nanocomposites incorporated with fillers have better viability than unfilled ones; the decreased viability of the blank sample may be a result of using the chemical crosslinker glutaraldehyde. It is reported that chemical crosslinking may have a negative effect on cell proliferation^51^.

According to the ISO 10993-5 in-vitro cytotoxicity standard, a cytotoxic effect is defined as a "reduction of cell viability by more than 30%"^52,53^.

Figure S7 represents a comparison between the 2 fillers at different concentrations. It can be noticed that the nanocomposites containing 2 and 5 wt% of LP demonstrate high viability of cells (more than 70%), which is considered safe for normal skin fibroblast. However, for the nanocomposites containing 2 and 5 wt% of Cc, Cell vitality values reached the minimum ISO requirements (70.5 and 69.07), which is the border between cytotoxic and non-cytotoxic.

As shown in Tables 2 and 3, the nanocomposites containing 10 and 15 wt% LP and Cc show high toxicity, reaching 34.2, 40.2 for 10, 15% LP and 55.8, 59.9% for 10 and 15% Cc, respectively, at 125 µg/ml.

### Antimicrobial analysis

#### Shake flask method (dynamic method)

The antimicrobial activity of PVA/starch blank and nanocomposites containing 5 wt% of each filler was chosen as the optimum filler concentration to be tested against 3 selected microorganisms Gram-negative bacteria, *Escherichia* coli, Gram-positive bacteria *Staphylococcus aureus* and *candida albicana*. The results are presented in Table 4. As indicated in the table, PVA/starch blank film did not show any antimicrobial activity against tested microorganisms. On the other hand, both types of nanocomposites positively affect the tested organisms, and higher values were recorded for nanocomposites containing LP.

**Table 4:** Relative reduction [OD reduction (%)] of the pathogenic strains after 24 h incubation using the shake flask method.

Tested microorganism	(%) CFU reduction		
	PVA/Starch	PVA/starch/5 wt%LP	PVA/starch/5 wt%Cc
*Escherichia coli*	0	76.86	65.22
*Staphylococcus aureus*	0	75.10	66.26
*Candida albicans*	0	76.53	54.93

The antimicrobial potency of these nanocomposites is believed to be due to phenolic compounds, flavonoids, and essential oils contained in the bio-fillers, which added biologically active values and consequently enhanced the antimicrobial activities of the nanocomposites.

## Conclusion

In this study, nanocomposites based on PVA/starch incorporated with two different types of waste bio-fillers namely *Citrus limon* (peels) and *Citrullus colocynthis* (seeds and shells) powders have been prepared using solution casting technique. The target was to get rid of such waste in one hand and on the other hand to obtain eco-friendly composites useful for various applications such as biomedical and or anti static applications. The dielectric properties and water resistance improved after the incorporation of both fillers. Considering the testing findings, PVA/starch/LP composites were more water resistant.

Nanocomposites containing higher concentrations of the biofillers (10 and 15 wt.%) show cytotoxic effects on normal fibroblast cell lines, so they are excluded in biomedical applications, but they still have advantages in use in insulating applications as described by the dielectric results.

In a final conclusion, Lemon peels and *Citrullus colocynthis* powders in the 5 wt% filler ratio act as antimicrobial and reinforcing agents for the PVA/starch matrix. Nevertheless, the nanocomposites containing lemon peels were preferable in biomedical applications. They recommended undergoing an in vivo study to confirm their efficiency for intended use as skin contact material as patches or wound healing material. On the other hand, the values of the σ dc are in the order of 10^−10^ S/cm which highly recommends such composites to be used in the insulating purpose that wide up the applications of such composites.

### Supplementary Information

Supplementary Figures.

## Data Availability

All data generated or analyzed during this study are included in this published article [and its supplementary information files].
