# Author Correction: Novel nano composites from *Citrus limon* and *Citrullus colocynthis* agricultural wastes for biomedical applications

**DOI:** 10.1038/s41598-024-70323-8

**Published:** 2024-08-21

**Authors:** Nagwa A. Kamel, D. A. Wissa, Salwa L. Abd‑El‑Messieh

**Affiliations:** 1 Microwave Physics and Dielectrics Department, Physics Research Institute, National Research Centre, Giza, Egypt; 2 Solid State Physics Department, Physics Research Institute, National Research Centre, Giza, Egypt

Correction to: *Scientific Reports* 10.1038/s41598-024-67423-w, published online 28 July 2024

The original version of this Article contained an error in the Abstract, where the ‘Human Normal Fibroblast cell lines (HSF)’ was incorrectly misspelt as ‘(HFB4)’.

As a result, in the Abstract section

“Additionally, the biocompatibility of the composites on human normal fibroblast cell lines (HFB4) was tested using MTT (3-[4,5-dimethylthiazol-2-yl]-2,5 diphenyl tetrazolium bromide) assay.”

now reads

“Additionally, the biocompatibility of the composites on human normal fibroblast cell lines (HSF) was tested using MTT (3-[4,5-dimethylthiazol-2-yl]-2,5 diphenyl tetrazolium bromide) assay.”

Furthermore, the Supplementary Information file ‘Supplementary Figures’ contained an error in the Author Information section.

The incorrect version of ‘Supplementary Figures’ appears below.

The Supplementary Information file has been updated.

The original Article has been corrected.

### Supplementary Information

Supplementary Figures.

