# Supplementary material for: Novel nano composites from Citrus limon and Citrullus colocynthis agricultural wastes for biomedical applications
Source: Sci Rep. 2024 Jul 28;14:17343. doi: 10.1038/s41598-024-67423-w (PMC11284205; doi:10.1038/s41598-024-67423-w)
Supplement: Supplementary file 1 — Supplementary Figures. [file 41598_2024_67423_MOESM1_ESM.doc]

Novel nano composites from Citrus limon and Citrullus colocynthis agriculture wastes for biomedical applications

Nagwa A.Kamel^a*^ , D. A. Wissa ^b^ , Salwa L. Abd-El-Messieh ^a^.

1. Microwave Physics and Dielectrics Department, Physics Research Institute, National Research Centre, Giza, Egypt.
   b. Solid State Department, Physics Research Institute, National Research Centre, Giza, Egypt.

Corresponding author: Nagwa A.Kamel

[nagwakamel@gmail.com](mailto:nagwakamel@gmail.com)

[Na.kamel@nrc.sci.eg](mailto:Na.kamel@nrc.sci.eg)

| 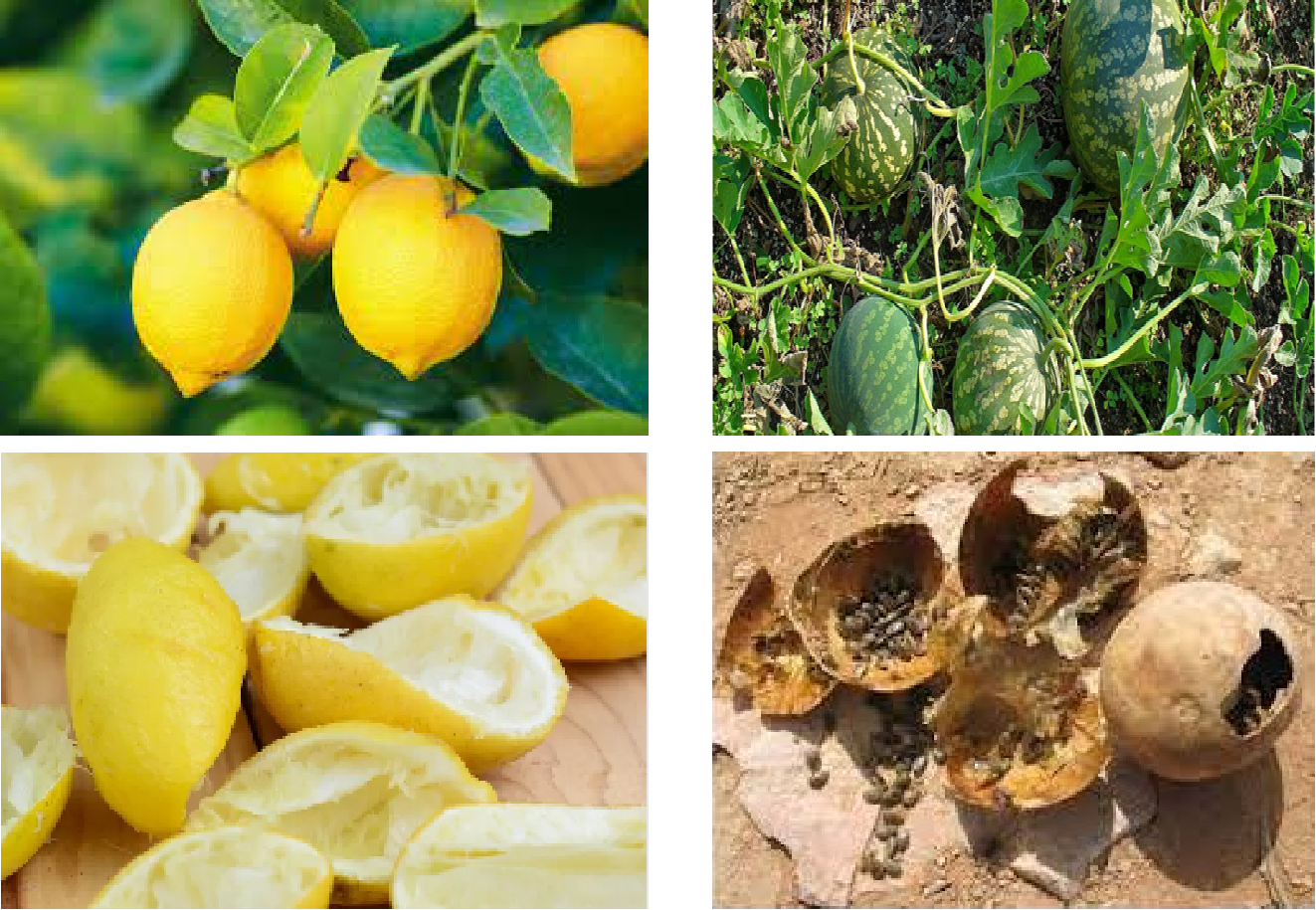 | |
| --- | --- |
| **Citrus limon** | **Citrullus colocynthis** |
| **Fig. S1.** Representation of the used fillers | |


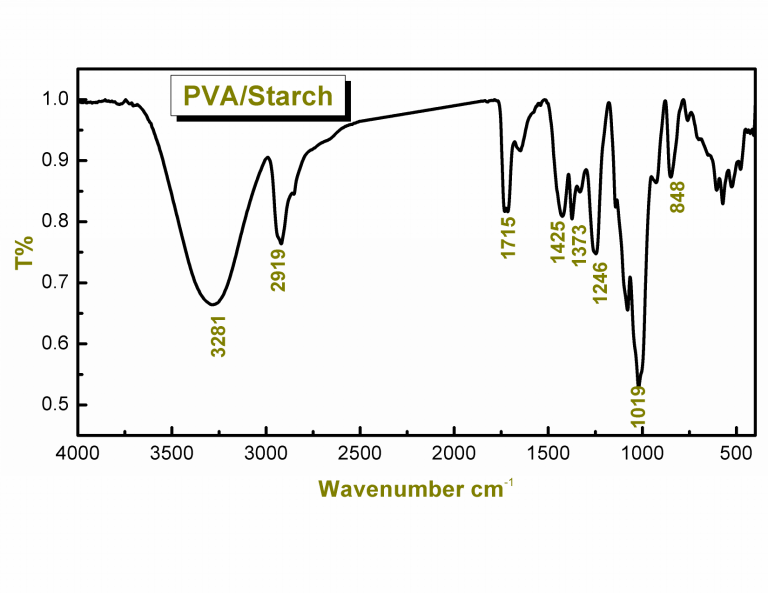


**Fig.S2.**  FTIR spectrum of blank (PVA /starch)


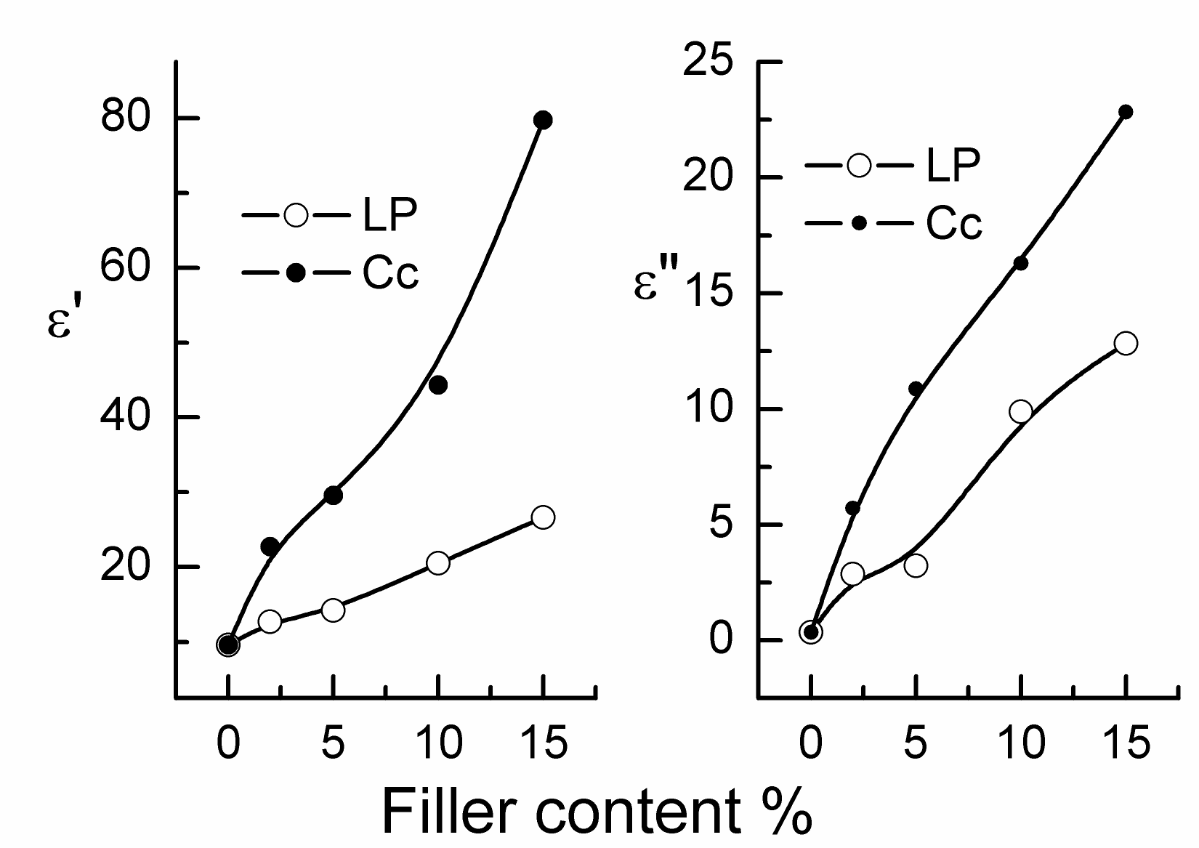


**Fig S3.** ε′ and ε″ V.s filler content at f = 100 kHz

| 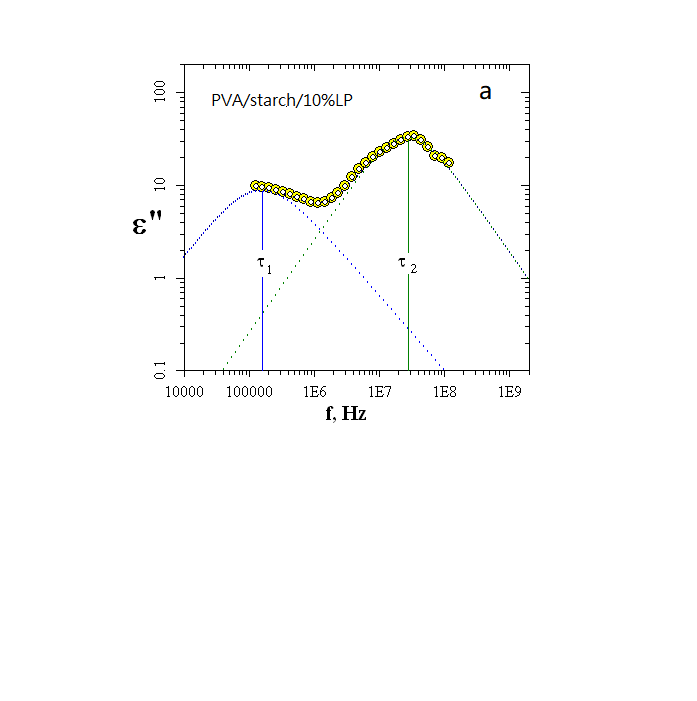 |
| --- |
| 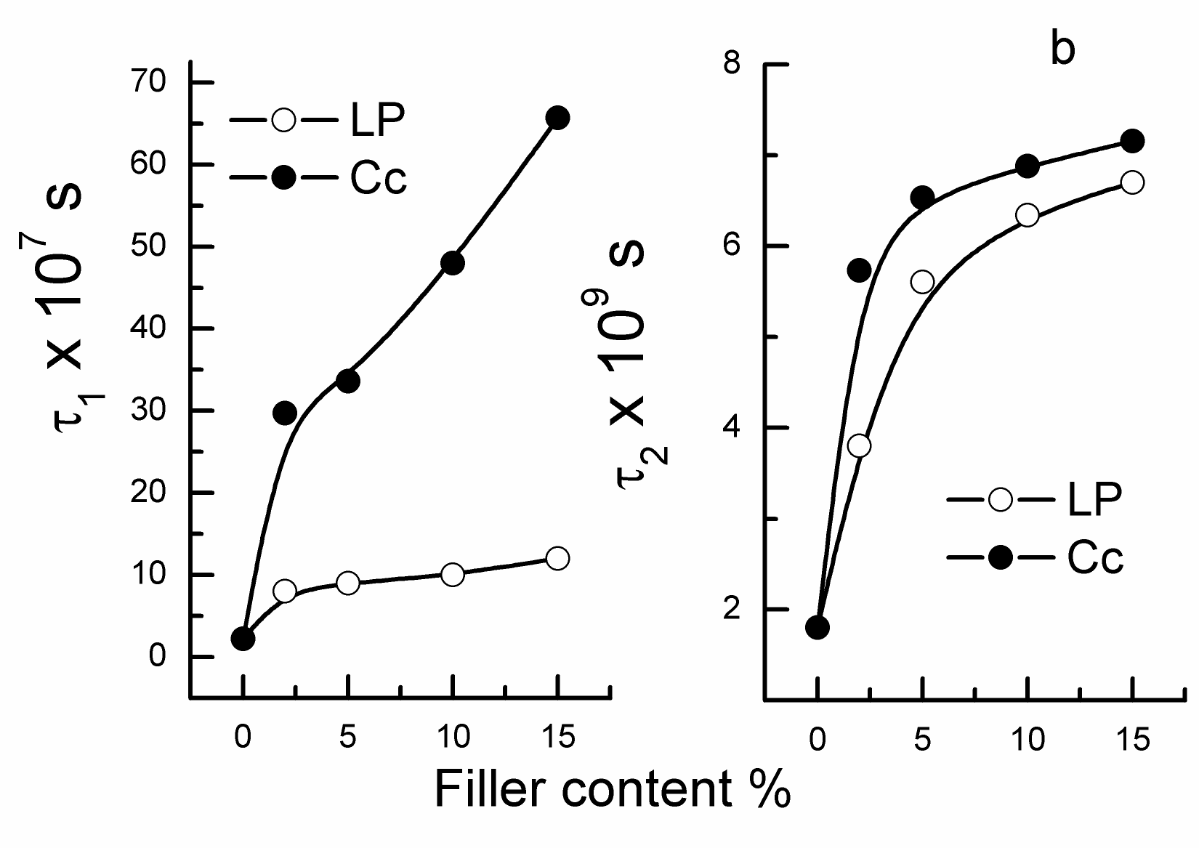 |
| **Fig S4.** (a) Example of the analyses for PVA/10%LP (b )τ_1_ and τ_2_ versus filler content |

| 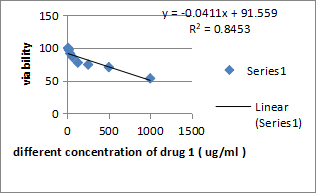  a |
| --- |
| 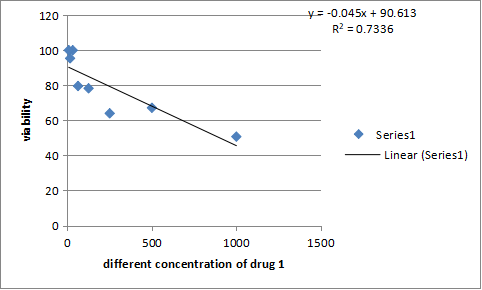  b |
| **Fig.S5.** Viability versus different concentrations for (a)PVA/starch/2%LP and (b) PVA/starch/5%LP |

| 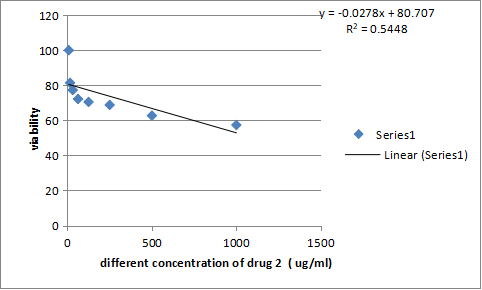  a |
| --- |
| 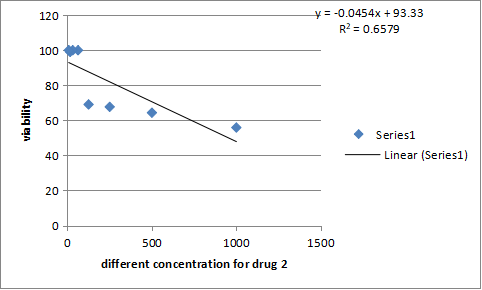  b |
| **Fig. S6.**Viability versus different concentration of (a)PVA/starch/2wt%Cc and (b)PVA/starch/5wt%Cc |

**
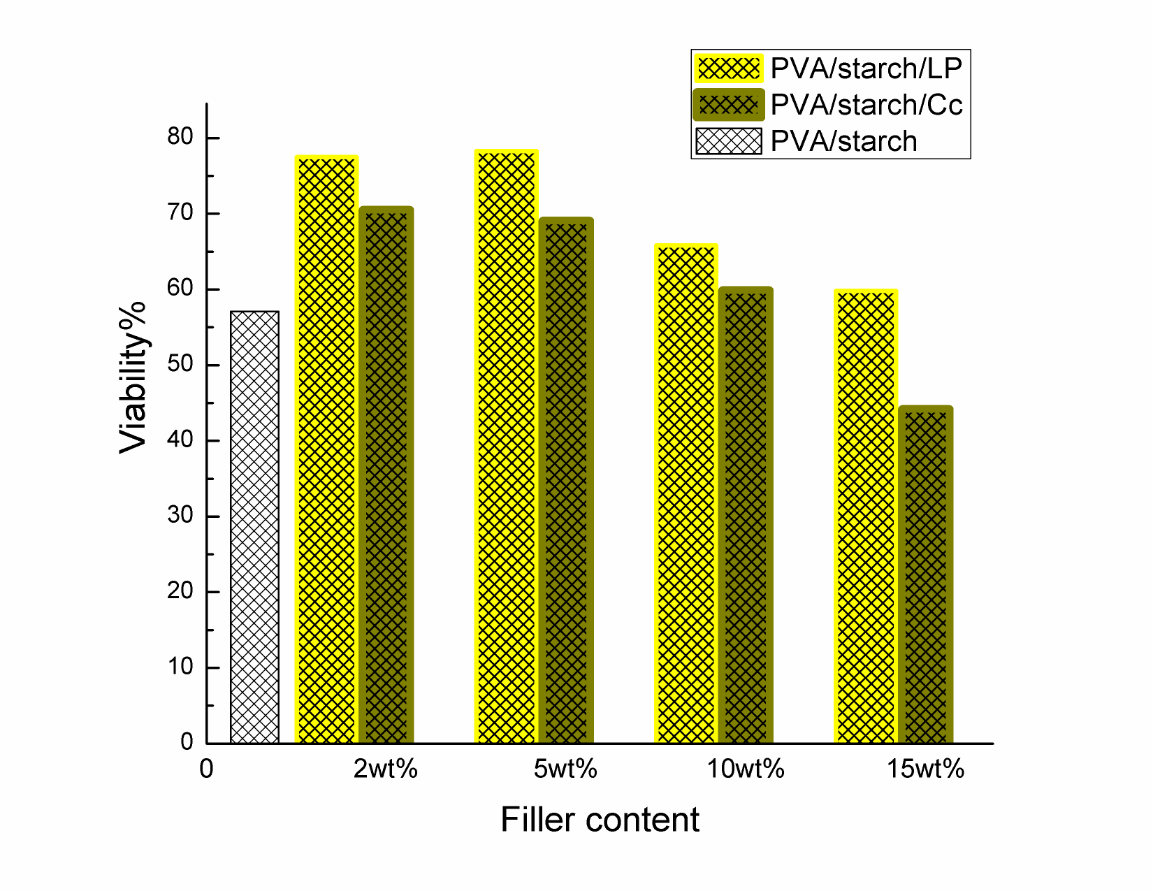
**

**Fig.S7.** Comparison between the viability% of the PVA/starch nanocomposites loading LP and Cc at (125ug /ml) drug concentration.
